# Reliability of a dried urine test for comprehensive assessment of urine hormones and metabolites

**DOI:** 10.1186/s13065-021-00744-3

**Published:** 2021-03-15

**Authors:** Mark Newman, Desmond A. Curran

**Affiliations:** Precision Analytical Inc., 3138 NE Rivergate Street #301C, Mcminnville, OR 97128 USA

**Keywords:** Dried urine testing, GC–MS/MS, LC–MS/MS, Reproductive hormones, Estrogen, Testosterone, Androgens, Organic acids

## Abstract

**Background:**

Mass spectrometry allows for analysis of multiple hormone and organic acid metabolites from small urine volumes; however, to assess the full extent of daily hormone production, 24-h urine collections are usually required. The aims of this study were, first, to confirm that mass spectrometric analysis of an array of hormones and organic acids would yield similar results in both liquid and dried urine, and, second, to determine if collection of four dried spot urine samples could be substituted for a 24-h collection when measuring reproductive hormones.

**Methods:**

Two study populations were included in this prospective observational study. Twenty individuals collected both a spot liquid urine and dried urine on filter paper to analyze eight organic acids. A second group of 26 individuals collected both a 24-h urine and four dried spot urines during waking hours throughout the same day for evaluation of 17 reproductive hormones and metabolites; data from 18 of these individuals were available to compare liquid versus dried urine results. Dried urine was extracted, hydrolyzed, and derivatized before analysis by mass spectrometry; all analytes from dried urine were normalized to urine creatinine.

**Results:**

Reproductive hormone results from dried and liquid urine were in excellent agreement with intraclass correlation coefficients (ICCs) greater than 0.90; comparison of dried to liquid urine for organic acids showed good to excellent agreement (ICC range: 0.75 to 0.99). Comparison between the 4-spot urine collection and 24-h urine collection methods showed excellent agreement (ICC > 0.9) for 14 of the 17 urine metabolites and good agreement for the others (ICC 0.78 to 0.85) with no systematic differences between the two methods of collection.

**Conclusions:**

The burden of urine collection can be reduced using collection of four spot dried urines on filter paper without compromising comparability with hormone results from a 24-h urine collection. A large number of urine analytes can be assessed from the dried urine with similar results to those from liquid urine. Given the ease of sample handling, this 4-spot dried urine assay would be useful for both clinical assessment of patients and for large epidemiologic studies.

**Supplementary information:**

The online version contains supplementary material available at 10.1186/s13065-021-00744-3.

## Introduction

Analysis of an array of hormones or metabolites may be useful for the clinician faced with a patient with a multitude of nonspecific symptoms that lead to a large differential of diagnoses, for screening of multiple diseases at once, for a patient on hormonal replacement therapy [1, 2], or for any patient where a complete picture of production and metabolism of a hormonal pathway is required [3]. Measurement of steroid hormones in urine can be an essential component to the diagnosis of hormone-related disorders [4, 5]. Some patients may require sampling over multiple days to determine monthly variations or effects of change in treatment. For hormones with known circadian or pulsatile fluctuations, a representation of the entire day is essential [6]; however, collection and storage of a 24-h urine can be cumbersome for patients [7, 8].

Mass spectrometry technology, both liquid chromatography tandem mass spectrometry (LC–MS/MS) for water-soluble compounds and gas chromatography tandem mass spectrometry (GC–MS/MS) for non-polar compounds, is now routinely used to measure arrays of steroid hormones and organic acids because of its high assay sensitivity, accuracy with small volumes, and ability to evaluate multiple analytes at the same time [9, 10]. This methodology allows for a complete profile of urine reproductive hormonal metabolites and multiple organic acids with high resolution of closely related structures [10].

Collection of urine on filter paper, which can then be dried and stored at room temperature until received by the laboratory [11–13], offers a significant advance in patient convenience and may improve patient adherence. If multiple samples are collected throughout the day, there is potential to capture both the diurnal variation of hormones along with the full range of daily hormonal production [11, 14]. Our prior proof-of-concept papers have demonstrated that dried urine leads to equivalent measures as liquid urine in our hands for cortisol, cortisone, and the cortisol metabolites, ⍺-tetrahydrocortisol, β-tetrahydrocortisol, and tetrahydrocortisone [14] as well as for estrone, estradiol, ⍺-pregnanediol, and β-pregnanediol [11]. Others have found similar results between liquid and dried urine with organic acids [12, 15, 16]. The urinary analytes in this study included female and male reproductive hormones, 6-hydroxymelatoninsulfate, and a number of organic acids, which have a multitude of uses (Additional file 1: Table S1). Inclusion of multiple metabolites from the estrogen and androgen pathways allows for a complete picture of estrogen and testosterone production and metabolism [17, 18].

The primary goal of this study was to confirm that measurement of the urinary profile of reproductive hormones, 6-hydroxymelatoninsulfate, and an array of organic acids extracted from dried urine collected on filter paper as analyzed by tandem mass spectrometry would provide results in agreement with measurements from liquid urine. The secondary aim was to demonstrate that measurement of reproductive hormones in a collection of four dried urine samples over a 15-h span throughout the day would accurately reflect the measurements of these hormones in a 24-h urine collection.

## Methods

### Study populations

A prospective observational study of urine collected from a population of healthy adult volunteers who agreed to participate in validation of urine analyses was conducted. This first study population included 26 individuals who provided data on hormonal measures (cortisol and cortisol metabolites, reproductive hormones, and 6-hydroxymelatoninsulfate) to compare samples from the 4-spot urine collection method to a 24-h urine collection. A subset of these individuals (n = 18) had data available to compare measures from dried versus liquid urine. As cortisol and cortisol metabolites [14] and ⍺ and β-pregnanediol [11] were validated in previous analyses, only the following hormones were included in this analysis: estrone (E1), estradiol (E2), estriol (E3), 2-hydroxyestrone (2OHE1), 2-hydroxyestradiol (2OHE2), 4-hydroxyestrone (4OHE1), 16-hydroxyestrone (16OHE1), 2-methoxyestrone (2-methoxyE1), testosterone (T), epitestosterone (EpiT), 5⍺- dihydrotestosterone (DHT), androsterone, etiocholanolone, 5⍺-androstanediol, 5β-androstanediol, dehydroepiandrosterone (DHEA), and 6-hydroxymelatoninsulfate. The data were collected between February and November of 2015 and informed consent was obtained from all participants.

The second analysis included 20 individuals whose deidentified data was pulled from the larger databank of 144,561 laboratory visits. Each of these samples included a single first-morning urine collection to compare results of dried versus liquid urine for the following organic acids: homovanillic acid (HVA), vanillylmandelic acid (VMA), kynurenic acid, xanthurenic acid, methylmalonic acid (MMA), pyroglutamic acid, 5-hydroxyindoleacetic acid (5-HIAA), and β-hydroxyisovaleric (Hiv) acid.

All volunteers in both study populations reported no medical problems and were not pregnant. Individuals were not excluded based on current or recent use of any hormonal medications, as the goal was only to compare measurement values for differing methodologies. Eighty percent of women in the first study population and all women in the second study population were premenopausal.

### Sample collection

The 4-spot method involves urine samples collected at home at four times during the day: (1) the first urine of the day, (2) 2 h after awakening, (3) in the afternoon (approximately 4 PM), and 4) before bed (10PM). Participants collected samples by completely saturating 2 × 3 inches of filter paper (Whatman Body Fluid Collection Paper; Sigma-Aldrich, St. Louis, MO, USA) with urine. The paper was left exposed at room temperature for 24 h to dry. The stability of analytes in dried urine at room temperature for as long as 84 days has previously been demonstrated by this laboratory [11]. Dried samples were stored at − 80 °C until analyzed. Reproductive hormones were assessed in all four samples collected, while only the first morning sample was used for 6-hydroxymelatoninsulfate and the organic acid tests.

During the same day, all liquid urine samples for the 24-h collection were added to a low-density polyethylene plastic container (ES Robbins, Muscle Shoals, AL, USA) container with approximately 1 g of boric acid (Sigma-Aldrich, St. Louis, MO, USA) and kept refrigerated for the duration of the collection. The four dried urine samples removed a total of about 8 mL of urine from the 24-h collection. This was considered negligible and was not accounted for. The total volume of urine from 24-h collections was measured, and an aliquot was frozen and stored at − 80 °C until tested.

### Urine reproductive hormone analysis

The urinary steroid hormones were analyzed using proprietary in-house CLIA (Clinical Laboratory Improvement Amendments) approved assays on the Agilent 7890/7000B GC–MS/MS (Agilent Technologies, Santa Clara, CA, USA). All reagents, unless otherwise noted, were purchased from Sigma-Aldrich (St. Louis, MO, USA). A 600 ul aliquot of liquid urine was taken from the sample collection and the equivalent of approximately 600 ul of urine was extracted from the filter paper using 2 mL of 100 mM ammonium acetate adjusted to a pH of 5.9. These aliquots of the conjugated hormones were transferred to a C18 solid phase extraction (SPE) column (UCT LLC, Briston, PA, USA), eluted using methanol, and the eluate was dried under nitrogen at 40 °C.

The conjugated hormones were then hydrolyzed from their glucuronide and sulfate forms to free forms using enzymes from *Helix pomatia* (Sigma-Aldrich, St. Louis, MO, USA) in acetate buffer (55 °C, 90 min). The enzymatic reaction was quenched with sodium hydroxide and the hormones extracted with ethyl acetate. The ethyl acetate extracts were dried under nitrogen at 40 °C. The analytes were derivatized using a mixture of 100 ul acetonitrile (ACN) and 50 ul bis(trimethylsilyl)trifluoroacetamide (Sigma-Aldrich, St. Louis, MO, USA) for 30 min at 70 °C. Internal standards (Steraloids, Newport, RI, USA) were added prior to ethyl acetate extraction, and the percentage recovery from all assays was greater than 90%. Derivatized extract (1.6 ul) was injected into the GC–MS/MS. Samples and controls were analyzed along with a standard curve spanning the expected range of concentrations. Instrument conditions for the oven were an initial temperature of 130 °C increasing to 200 °C at 25 °C/min, then to 230 °C at 4.3 °C/min, and finally to 290 °C at 25 °C/min. Multiple reaction monitoring transitions for ion mass > ion product of fragmentation can be found in Table 1. Creatinine was measured using a conventional colorimetric (Jaffe) method, after initial extraction from the filter paper. The average inter-assay coefficient of variation was 6.7% for creatinine. In addition to expressing the measures per mg of creatinine to correct for variations in filter paper saturation and hydration status, a secondary equation was applied to reduce bias related to the effects of age, sex, weight, and height on creatinine excretion [19].

**Table 1:** Multiple reaction monitoring transitions for ion mass > ion product of fragmentation for urinary reproductive hormones

Analyte	Transition
Estrone (E1)	342.1 > 257.1
Estradiol (E2)	416.2 > 285.1
Estriol (E3)	504.3 > 296.3
2-Hydroxyestrone (2OHE1)	430.2 > 345.0
2-Hydroxyestradiol (2OHE2)	462.2.2 > 195.2
4-Hydoxyesrone (4OHE1)	430.2 > 354.0
16-Hydroxyestrone (16OHE1)	430.2 > 286.1
2-Methoxyestrone (2-methoxyE1)	372.2–342.1
Testosterone (T)	360.2 > 174.1
Epitestosterone (EpiT)	360.2 > 174.1
Dihydrotestosterone (DHT)	347.2 > 271.2
Androsterone	347.2 > 271.2
Etiocholanolone	347.2 > 253
5⍺-androstanediol	421.2 > 331.2
5β-androstanediol	241.2 > 185.2
Dehydroepiandrosterone (DHEA)	303.7 > 199.1

### Urine 6-hydroxymelatoninsulfate and organic acid analysis

The hydrophilic analytes were assessed by LC–MS/MS using proprietary in-house CLIA approved assays. For the 6-hydroxymelatoninsulfate assay, a 30 ul aliquot was taken from the methanol elution of both the liquid urine collection and the waking sample dried urine collected on filter paper. This extract was then reconstituted in 130 ul of deionized water. For the organic acids, a 100 ul aliquot of liquid urine was taken and an equivalent amount was extracted from the waking sample dried urine filter paper using 250 ul of water with the addition of 50 ul 100 mM ammonium acetate (Sigma-Aldrich, St. Louis, MO, USA) adjusted to a pH of 5.9 and 2% formic acid.

For 6-hydroxymelatoninsulfate, 20 ul was injected into an ultra-performance liquid chromatography (UPLC) (Waters Corporation, Milford, MA, USA) column with a Waters™ tandem quadrupole mass spectrometer detector (TQD). The sample was eluted from a 1.8u 2.1 × 50 mm pentafluorophenyl (PFP) column (Agilent Technologies, Santa Clara, CA, USA) using a gradient of 95% 0.001% formic acid in 5% ACN to 45% 0.001% formic acid in 55% ACN. For the organic acids, 5 ul was injected into the Waters™ UPLC column with TQD. These analytes were eluted from a 1.6 um 2.1 × 50 mm Luna Omega PS C18 column (Phenomenex, Torrance, CA, USA) using a gradient 99.9% 0.2% formic acid in 0.1% ACN to 73% 0.2% formic acid in 27% ACN. Multiple reaction monitoring transitions for ion mass > ion product of fragmentation for urine 6-hydroxymelatoninsulfate and the organic acids are listed in Table 2. The same creatinine corrections of the measures used for the reproductive hormones were also used for 6-hydroxymelatoninsulfate and the organic acids.

**Table 2:** Multiple reaction monitoring transitions for ion mass > ion product of fragmentation for urine 6-hydroxymelatoninsulfate and the organic acids

Analyte	Transition
6-Hydroxymelatoninsulfate	372.5 > 176.2
d4-6-Hydroxymelatoninsulfate	331.5 > 179.2
Homovanillic acid (HVA)	183.0 > 137.0
d2-Homovanillic acid (d2-HVA)	185.0 > 139.0
Vanillylmandelic acid (VMA)	197.0 > 138.0
d3-Vanillylmandelic acid (d3-VMA)	199.8 > 140.9
Kynurenic acid	190.0 > 144.0
d5-Kynurenic acid	192.0 > 149.0
Xanthurenic acid	206.0 > 160.0
d3-Xanthurenic acid	210.0 > 164.0
Methylmalonic acid (MMA)	117.0 > 73.0
d2-Methylmalonic acid (d2-MMA)	120.0 > 76.0
Pyroglutamic acid	130.0 > 84.0
d5-Pyroglutamic acid	135.0 > 89.0
5-Hydroxyindoleacetic acid (5-HIAA)	192.0 > 146.0

### Statistical methods

A sample size of 18 individuals provides a power of greater than 80% to detect an intraclass correlation coefficient (ICC) of at least 0.6 with an alpha of 0.05 [20]. The statistical analyses were performed using SAS/STAT^®^ software, Version 9.3 (SAS Institute Inc., Cary, NC, USA) and generated 2-sided p-values.

Variables are described as mean ± standard deviation if normally distributed and median (interquartile range (IQR)) if the distribution was skewed. Student’s *t* test (for normally distributed variables) or the Wilcoxon rank-sum test (for skewed variables) were used to determine differences between men and women. Spearman correlation coefficients (ρ) were used to determine interclass associations between variables.

Consistency between liquid versus dried urine measures and 4-spot versus 24-h collection methodology was assessed using intraclass correlation coefficients (ICC). ICCs, which range from 0 to 1 with proximity to 1 indicating better agreement, assess for agreement of a measure between two differing methodologies within individuals [21]. Skewed variables were log transformed to approximate a normal distribution prior to assessing ICCs. As 4-spot (ng/mg-Cr) and 24-h (ug/d) measures were expressed in differing units, sex-specific Z-scores ([individual measurement-mean]/standard deviation) were created to standardize the measures and allow for direct comparison. Comparisons of differences between measures within an individual were assessed using signed-rank tests (for skewed variables) or paired t-tests (for normally distributed variables). Because the hypotheses of this paper were intrinsically correlated, no adjustments were made for multiple comparisons.

## Results

### Study populations

Characteristics of the first study population (n = 26) are shown in Table 3. All of these individuals (58% female; 100% Caucasian) had data available for comparison of 4-spot versus 24-h urine samples for male and female reproductive hormones. A subset (10 female, 8 male) also had measurements to compare liquid versus dried urine samples for both the reproductive hormones and 6-hydroxymelatoninsulfate. Characteristics of the second study population (n = 20; 75% female; 80% Caucasian/10% Hispanic/10% Asian-Pacific Islander), data from whom were used to compare a single first morning collection of liquid versus dried urine samples for the organic acid tests, are provided in Table 4.

**Table 3:** Age and 24-h measures of reproductive hormones and 6-hydroxymelatoninsulfate of the first study population

Variable	All	Female (n = 15)	Male (n = 11)
Age (years)	36.8 ± 14.5	33.7 ± 7.8	37.8 ± 18
Estrone (ug/d)^a^	13.69 (6.32, 19.16)	18.75 (6.83, 23.68)	7.67 (6.26, 13.68)
Estradiol (ug/d)^`^	1.73 (0.80, 3.26)	3.17 (0.89, 4.19)	1.14 (0.70, 1.62)
Estriol (ug/d)^a^	6.82 (3.57, 12.80)	11.64 (6.14, 16.55)	5.56 (3.45, 6.91)
2-Hydroxyestrone (ug/d)^a^	3.33 (1.27, 6.24)	5.82 (0.77, 8.46)	2.26 (1.27, 4.03)
2-Hydroxyestradiol (ug/d)^a^	0.25 (0.11, 0.67)	0.58 (0.06, 0.81)	0.19 (0.11, 0.26)
4-Hydroxyestrone (ug/d)^a^	0.37 (0.19, 0.75)	0.67 (0.19, 0.99)	0.30 (0.16, 0.36)
16-Hydroxyestrone (ug/d)^a^	0.89 (0.42, 1.48)	1.40 (0.36, 1.58)	0.49 (0.42, 0.90)
2-Methoxyestrone (ug/d)^a^	2.27 (1.30, 3.80)	3.56 (1.38, 5.50)	1.63 (1.05, 2.35)
Testosterone (ug/d)^b^	9.15 (4.97, 51.92)	5.43 (4.51, 8.01)	61.40 (42.14, 97.70)
Epitestosterone (ug/d)^b^	7.23 (4.01, 11.23)	4.44 (1.18, 7.49)	14.94 (7.99, 16.80)
5a-Dihydrotestosterone (ug/d)^b^	5.53 (2.72, 11.59)	2.72 (1.33, 5.46)	12.60 (9.88, 17.47)
Androsterone (ug/d)^a^	1331.99 (854.41, 1940.29)	1032.77 (691.70, 1528.59)	2083.52 (1005.51, 2716.33)
Etiocholanolone (ug/d)^a^	811.74 (463.17, 1045.23)	568.56 (430.73, 853.81)	1020.79 (716.22, 1423.92)
5α-Androstanediol (ug/d)^b^	102.50 (54.83, 197.14)	58.72 (46.49, 97.50)	229.01 (143.30, 292.53)
5β-Androstanediol (ug/d)^a^	35.58 (14.05, 60.75)	15.83 (9.38, 52.69)	60.75 (20.69, 122.18)
DHEA (ug/d)	166.14 (80.45, 431.61)	136.42 (80.45, 317.83	351.32 (52.69, 732.63)
6-Hydroxymelatoninsulfate (ug/ml)	7.87 (5.93, 13.85)	7.48 (5.93, 8.43)	12.21 (5.11, 20.06)

Age presented as mean ± SD; hormone data presented as median (IQR) as these data were skewed

^a^ p < 0.05; ^b^ p < 0.01 for differences between males and females as assessed by the Wilcoxon rank-sum test

DHEA: dehydroepiandrosterone

**Table 4:** Age and measures of organic acids from an early morning spot urine collection of the second study population

Variable	All	Female (n = 15)	Male (n = 5)
Age (years)	34.4 ± 6.6	34.5 ± 6.6	34.0 ± 7.4
Methylmalonic acid (ng/mg-Cr)	0.18 (0.16, 0.25)	0.20 (0.16, 0.29)	0.16 (0.16, 0.18)
Homovanillic acid (ng/mg-Cr)^b^	0.52 ± 0.16	0.57 ± 0.14	0.35 ± 0.05
Vanillylmandelic acid (ng/mg-Cr)	0.41 ± 0.12	0.43 ± 0.12	0.36 ± 0.13
Kynurenic acid (ng/mg-Cr)	0.25 ± 0.08	0.26 ± 0.08	0.21 ± 0.05
Xanthurenic acid (ng/mg-Cr)	0.06 (0.05, 0.07)	0.06 (0.04, 0.07)	0.06 (0.05, 0.07)
5-HIAA (ng/mg-Cr)^a^	0.44 (0.40, 0.55)	0.47 (0.41, 0.58)	0.37 (0.36, 0.41)
Pyroglutamic acid (ng/mg-Cr)	4.06 ± 0.69	4.11 ± 0.78	3.90 ± 0.33
β-Hydroxyisovaleric acid (ng/mg-Cr)	0.27 ± 0.12	0.29 ± 0.13	0.21 ± 0.07

Data are presented as mean ± SD if normally distributed and median (IQR) if right-skewed

^a^p < 0.05; ^b^p < 0.01 for differences between males and females as assessed by the Student t-test or Wilcoxon rank-sum test

5-HIAA: 5-hydroxyindoleacetic acid

### Agreement between Liquid and Dried Urine Measures

**Fig. 1:**
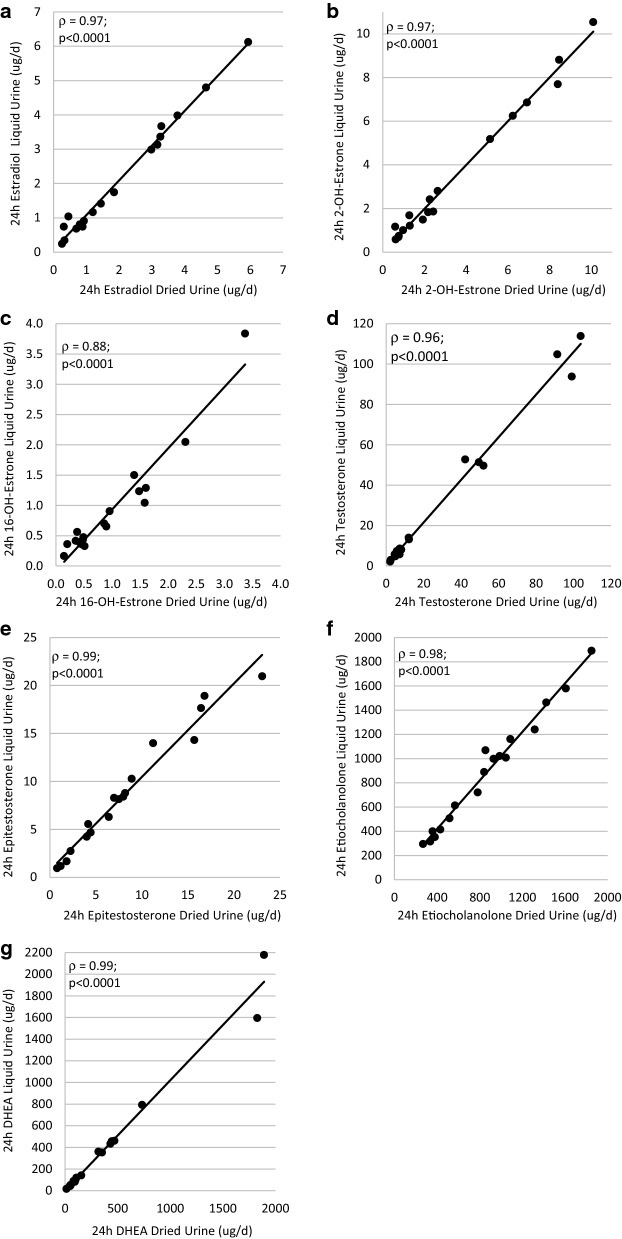
Correlations between the liquid versus dried measurements for select urine steroid hormones. The remainder are available in Additional file 1: Figure S1. Reported correlation coefficients are Spearman correlations. Cr: creatinine; DHEA: dehydroepiandrosterone

**Fig. 2:**
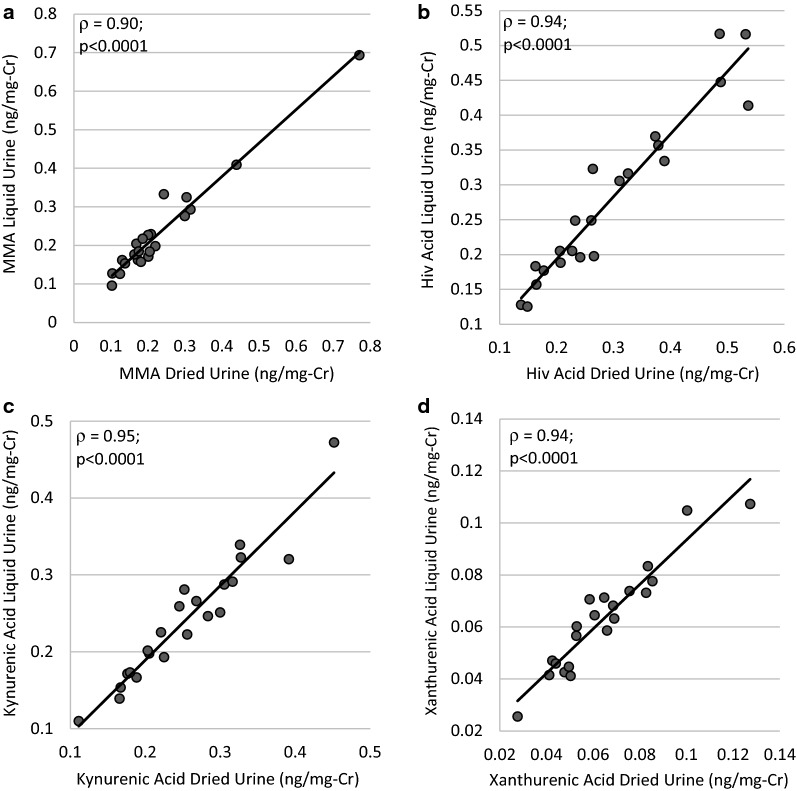
Correlations between the liquid versus dried measurements for select urine organic acids. The remainder are available in Additional file 1: Figure S2. Reported correlation coefficients are Spearman correlations. Cr: creatinine, Hiv: β-Hydroxyisovaleric

For the majority of analytes, there was excellent agreement (ICC > = 0.90) between the liquid and dried measures (Table 5). The exceptions were VMA (ICC = 0.79) and pyroglutamic acid (ICC = 0.75), which still had good agreement. Similarly, for the majority of analytes, there was no systemic directionality to the difference in the dried urine compared to the liquid urine. However, estrone and estriol were consistently higher when measured in liquid urine, while some of the organic acids – VMA, kynurenic acid, pyroglutamic acid, and β-hydroxyisovaleric acid – were more concentrated in the dried urine sample (Table 5). Representative interclass correlations (Spearman) between the two methods are shown in Fig. 1 and Fig. 2 (the rest are available in Additional file 1: Figure S1).

**Table 5:** Comparison of liquid versus dried urine analytes (n = 18)

Variable	Dried	Liquid	Difference (95% CI)	ICC (95% CI)
Hormones (n = 18)				
Estrone (ug/d)	10.78 (6.26, 18.73)	12.78 (7.22, 20.65)	1.28 [0.09, 2.47]^a^	0.92 [0.80, 0.97]
Estradiol (ug/d)	1.32 (0.70, 3.26)	1.29 (0.74, 3.36)	0.09 [-0.01, 0.19]	0.96 [0.89, 0.98]
Estriol (ug/d)	6.82 (3.45, 11.64)	7.15 (4.76, 14.41)	0.86 [0.05, 1.67]^a^	0.90 [0.77, 0.96]
2-Hydroxyestrone (ug/d)	2.22 (0.98, 6.24)	1.84 (1.17, 6.24)	− 0.01 [− 0.18, 0.16]	0.98 [0.94, 0.99]
2-Hydroxyestradiol (ug/d)	0.18 (0.09, 0.58)	0.18 (0.08, 0.47)	0.00 [− 0.02, 0.03]	0.97 [0.92, 0.99]
4-Hydroxyestrone (ug/d)	0.31 (0.16, 0.67)	0.40 (0.25, 0.62)	0.03 [− 0.02, 0.09]	0.95 [0.87, 0.98]
16-Hydroxyestrone (ug/d)	0.69 (0.42, 1.48)	0.61 (0.39, 1.24)	− 0.07 [− 0.18, 0.05]	0.95 [0.87, 0.98]
2-Methoxyestrone (ug/d)	1.64 (1.05, 3.56)	1.70 (1.50, 3.58)	0.12 [− 0.07, 0.30]	0.97 [0.94, 0.99]
Testosterone (ug/d)	7.64 (4.97, 49.46)	8.42 (5.80, 51.40)	2.07 [− 0.26, 4.40]	0.99 [0.98, 1.00]
Epitestosterone (ug/d)	7.23 (4.01, 11.23)	8.21 (4.23, 13.98)	0.51 [− 0.06, 1.07]	0.99 [0.97, 1.00]
5a-Dihydrotestosterone (ug/d)	5.53 (2.72, 11.59)	4.94 (2.68, 10.35)	− 0.61 [− 1.02, − 0.19]^a^	0.99 [0.97, 1.00]
Androsterone (ug/d)	1331.99 (929.43, 1933.30)	1237.63 (813.31, 1847.10)	− 33.02 [− 105.70, 39.67]	0.99 [0.98, 1.00]
Etiocholanolone (ug/d)	847.93 (430.73, 1088.18)	943.49 (414.13, 1161.61)	19.76 [− 13.02, 52.54]	0.99 [0.98, 1.00]
5α-Androstanediol (ug/d)	110.09 (57.50, 197.14)	119.40 (65.63, 226.93)	15.67 [− 12.67, 44.01]	0.90 [0.76, 0.96]
5β-Androstanediol (ug/d)	35.58 (14.05, 72.42)	46.39 (14.72, 65.39)	− 0.54 [− 13.93, 12.85]	0.88 [0.73, 0.95]
DHEA (ug/d)	131.42 (52.69, 444.35)	130.86 (53.27, 457.05)	8.65 [− 36.66, 53.96]	1.00 [0.99, 1.00]
6-Hydroxymelatoninsulfate (ug/d)	7.87 (5.93, 14.33)	8.26 (5.93, 14.67)	0.04 [− 1.14, 1.21]	0.99 [0.98, 1.00]
Organic Acids (n = 20)				
Methylmalonic acid (ng/mg-Cr)	0.18 (0.15, 0.23)	0.18 (0.16, 0.25)	0.00 [− 0.01, 0.02]	0.96 [0.90, 0.98]
Homovanillic acid (ng/mg-Cr)	0.52 ± 0.17	0.52 ± 0.16	− 0.01 [− 0.05, 0.02]	0.88 [0.74, 0.95]
Vanillylmandelic acid (ng/mg-Cr)	0.45 ± 0.13	0.41 ± 0.12	− 0.05 [− 0.08, -0.02]^a^	0.79 [0.57, 0.91]
Kynurenic acid (ng/mg-Cr)	0.26 ± 0.08	0.25 ± 0.08	− 0.01 [− 0.02, − 0.00]^a^	0.95 [0.88, 0.98]
Xanthurenic acid (ng/mg-Cr)	0.06 (0.05, 0.08)	0.06 (0.05, 0.07)	− 0.00 [− 0.00, 0.00]	0.98 [0.95, 0.99]
5-HIAA (ng/mg-Cr)	0.46 (0.43, 0.57)	0.44 (0.40, 0.55)	− 0.01 [− 0.05, 0.02]	0.98 [0.96, 0.99]
Pyroglutamic acid (ng/mg-Cr)	4.42 ± 0.72	4.06 ± 0.69	− 0.41 [− 0.58, − 0.23]^b^	0.75 [0.51, 0.89]
β-Hydroxyisovaleric acid (ng/mg-Cr)	0.29 ± 0.12	0.27 ± 0.12	− 0.02 [− 0.03, − 0.00]^a^	0.95 [0.88, 0.98]

For liquid and dried measurements, data presented as mean ± SD for normally distributed variables and median (IQR) for skewed variables; the differences were normally distributed and are presented as mean [95% CI]

^a^p < 0.05; ^b^p < 0.0001 for the liquid minus dried difference by paired t-test

5-HIAA: 5-hydroxyindoleacetic acid; Cr: creatinine, DHEA: dehydroepiandrosterone; ICC: intraclass correlation coefficient

### Agreement between the DUTCH 4-spot and 24-h urine collection for hormonal measures

**Fig. 3:**
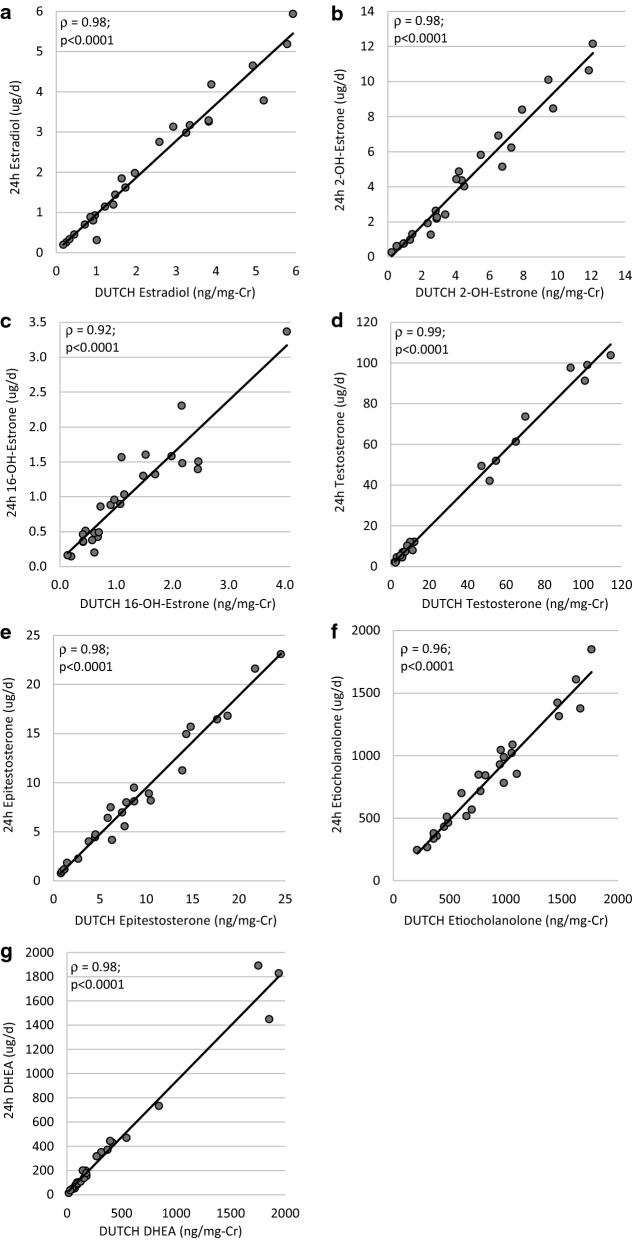
Correlations between the 24-h urine collection and 4-spot (DUTCH) urine collection measurements. The remainder are available in Additional file 1: Figure S3. Reported correlation coefficients are Spearman correlations. Cr: Creatinine, DHEA: dehydroepiandrosterone, DUTCH: Dried Urine Testing for Comprehensive Hormones

The measurement of reproductive hormones in urine samples collected four times throughout the day were comparable to the gold standard of a 24-h urine collection (Table 6). As the measures from the 4-spot urine collection are reported in ng/mg-Cr and the 24-h urine measures are reported in ug/d, sex-specific Z-scores were created for direct comparison of the two methodologies. There was excellent consistency (ICC > 0.9) for the majority of the analytes and good consistency for the remainder (estriol, 5⍺ and 5β-androstanediol) (Table 6). There was no systematic directionality to the differences between any of the Z-scores (Table 6). Representative interclass (Spearman) correlations between the analytes are shown in Fig. 3 (the remainder are shown in Additional file 1: Figure S3).

**Table 6:** Comparison of the urinary hormone profile using the 4-spot (DUTCH) or 24-h urine collection method (n = 26)

Variable	24-h urine collection (ug/d)	4-spot (ng/mg-Cr)	Z-score difference [95% CI]^a^	ICC [95% CI]
Estrone	13.69 (6.32, 19.16)	14.37 (7.13, 18.75	0.00 [− 0.13, 0.13]	0.95 [0.89, 0.97]
Estradiol	1.73 (0.80, 3.26)	1.68 (0.92, 3.81)	0.00 [− 0.16, 0.16]	0.92 [0.83, 0.96]
Estriol	6.82 (3.57, 12.80)	7.39 (4.15, 12.96)	0.00 [− 0.24, 0.24]	0.82 [0.64, 0.91]
2-Hydroxyestrone	3.33 (1.27, 6.24)	3.72 (1.45, 6.75)	− 0.00 [− 0.09, 0.09]	0.97 [0.94, 0.99]
2-Hydroxyestradiol	0.25 (0.11, 0.67)	0.30 (0.13, 0.49)	− 0.00 [− 0.10, 0.10]	0.97 [0.93, 0.99]
4-Hydroxyestrone	0.37 (0.19, 0.75)	0.45 (0.27, 0.81)	− 0.00 [− 0.15, 0.15]	0.93 [0.86, 0.97]
16-Hydroxyestrone	0.89 (0.42, 1.48)	0.93 (0.57, 1.69)	0.00 [− 0.19, 0.19]	0.90 [0.77, 0.95]
2-Methoxyestrone	2.27 (1.30, 3.80)	2.11 (1.54, 4.15)	0.00 [− 0.19, 0.19]	0.90 [0.78, 0.95]
Testosterone	9.15 (4.97, 51.92)	9.33 (5.36, 54.73)	− 0.00 [− 0.10, 0.10]	0.97 [0.93, 0.98]
Epitestosterone	7.23 (4.01, 11.23)	7.53 (3.81, 13.90)	− 0.00 [− 0.07, 0.07]	0.99 [0.97, 0.99]
5a-Dihydrotestosterone	5.53 (2.72, 11.59)	6.40 (2.16, 11.91)	− 0.00 [− 0.09, 0.09]	0.97 [0.94, 0.99]
Androsterone	1331.99 (854.41, 1940.29)	1346.46 (818.59, 2065.66)	− 0.00 [− 0.09, 0.09]	0.97 [0.94, 0.99]
Etiocholanolone	811.74 (463.17, 1045.23)	797.60 (475.43, 1061.66)	− 0.00 [− 0.09, 0.09]	0.98 [0.95, 0.99]
5α-Androstanediol	102.50 (54.83, 197.14)	86.74 (51.31, 200.70)	− 0.00 [− 0.27, 0.27]	0.78 [0.57, 0.89]
5β-Androstanediol	35.58 (14.05, 60.75)	34.49 (13.91, 62.77)	0.00 [− 0.22, 0.22]	0.85 [0.70, 0.93]
DHEA	166.14 (80.45, 431.61)	162.85 (78.17, 394.62)	− 0.00 [− 0.05, 0.05]	0.99 [0.99, 1.00]

The ICCs were calculated between measurements expressed as standardized sex-specific Z-scores (mean ± SD for all = 0.00 ± 0.98). Data are presented as median (IQR) for measurements and the difference (24-h minus 4-spot) in individual Z-scores is presented as mean [95% confidence interval]

^a^All p-values > 0.9 by paired t-test

Cr: creatinine; DHEA: dehydroepiandrosterone; ICC: intraclass correlation coefficient

A sensitivity analysis to verify the need for creatinine correction was done by calculating ICCs for the agreement between sex-specific Z-scores from the 24-h measures and the 4-spot assay without correction for creatinine. Without the creatinine correction, the ICCs were all lower than those observed with the creatinine correction (with the exception of estriol which had a slightly higher ICC by 0.04) with the degree of difference ranging from − 0.04 to 0.15 and averaging 0.07. An example of the interclass correlations (Spearman) between the 24-h measures and the 4-spot assay with and without the creatinine corrections is shown in Additional file 1: Figure S4.

## Discussion

This study demonstrated the feasibility of accurately measuring multiple (up to 32) analytes in dried urine samples collected on filter paper using assays that conform to CLIA criteria. All measurements from dried urine demonstrated at least good agreement with measures from liquid urine, and the majority (83%) demonstrated excellent agreement with intraclass correlations greater than 0.9. For most analytes, neither loss nor excess concentration occurred during the sample drying or laboratory extraction process. In addition, measurement of the reproductive steroid hormones, which are usually evaluated from a 24-h collection due to their pulsatile release [22], were well represented by the 4-spot dried urine collection with at least good agreement of the 4-spot measurement with the 24-h gold standard measure for all steroid metabolites and excellent agreement (> 0.9) for the majority (82%). There were no systematic differences between the relative amount of hormone collected by either methodology. The 4-spot dried urine (DUTCH) methodology allows for efficient, accurate assessment of numerous urine metabolites using a convenient collection method, while avoiding the need for a full 24-h liquid urine collection.

The 4-spot dried urine collection has previously been shown to be representative of select 24-h measures of steroidal hormones by our group [11], as measurement of urinary ⍺-pregnanediol, β-pregnanediol, estrone, and estradiol with this method are representative of both 24-h urine collections as well as serum hormone concentrations. In fact, repeated assessments over a month demonstrated that the dried urine collections could accurately recreate the changes observed in serum during the menstrual cycle [11]. In addition, not only does the 4-spot urine method accurately represent a 24-h urine collection for urinary cortisol, cortisone, and cortisol metabolites, but it can also be used to represent the expected diurnal pattern observed with salivary measures, if each of the four collections is considered individually [14].

Previous studies have validated the usefulness of high throughput GC–MS/MS for urine steroid profiling of more than 30 metabolites; however, this was done in liquid urine [5]. Conversely, measurement of organic acids in spot dried urine samples using filter paper has already been well-validated as a technique for screening neonates for metabolic disorders [15, 23, 24] and for screening for neuroblastoma [25] with similar recoveries obtained from liquid and dried urine. Dried urine has also been used to measure other urine analytes of interest, such as sodium and potassium, with similarly high levels of stability [26]. As with others who used either filter paper or cotton swabs [27], we found that dried urine results are in agreement with liquid urine results, reduced the burden on patients, and had good stability over time [11]. Still, agreement between 24-h urine collections and early-morning, single spot urine collections for hormonal analysis are often poor [28]. This study now extends our prior findings [11, 14] to show that the increase to four spot urines spaced throughout the waking hours provides better coverage of the hormonal output for all the male and female reproductive hormones and metabolites, resulting in strong agreement with 24-h urine measures.

There are some caveats that must be considered when interpreting our results. During the urine collection, there may have been differences in saturation of the filter paper. Expressing the analyte concentration per mg of creatinine is designed to address this, while also correcting for hydration. The method used for creatinine adjustment also accounts for differences in creatinine excretion related to body size. This does rely on accurate self-reporting of age, height, and weight by the participants, so an estimate of expected creatinine excretion can be made. This may lead to the introduction of inaccuracies due to misreporting of individual characteristics; however, our statistical sensitivity analyses indicated that the DUTCH measurements were in better agreement with the 24-h urine results with the application of the creatinine corrections (see Additional file 1: Figure S4), with the ICC for some metabolites increasing by as much as 0.15 and raising the level of agreement with 24-h measures from good to excellent.

One of the limitations of this 4-spot dried urine method is that reference ranges are laboratory-specific and non-standardized. Still, interpretation of values above and below these reference ranges should be similar to that of other assays. In this study, there was some loss of hormone for estrone and estriol with the filter paper methodology, which may be related to differences in extraction efficiency between the steroid conjugates and creatinine, loss during the drying process, or incomplete saturation of the filter paper. Still, the difference was less than 12% of the total and would be compensated for by an adjustment of the reference range. In addition, we have previously shown that the dried urine measure of estrone has clinical utility because it is representative of serum estrogen measurements [11]. A number of organic acids plus DHT were more concentrated in the dried urine samples, on average. This may be due to differences in extraction efficiency, a matrix effect or analyte concentration during the drying process; however, this difference did represent less than 10% of the sample. Fortunately, due to the high level of agreement between the Z-scores from the DUTCH methodology and 24-h collections, laboratory reference ranges should account for these differences. Another issue is there are known genetic differences in glucuronidation of testosterone that may impact relative metabolism and urinary concentrations of testosterone and epitestosterone [29], and this may mean urinary androgen measures are not fully representative of production rates in a small percentage of individuals. There are also genetic differences in the enzymes that metabolize estrogens, potentially shifting the ratio of 2-hyroxylation to 16-hydroxylation metabolites [30], but these differences may be clinically relevant and indicative of cancer risk [31]. Our study population had limited diversity and was primarily Caucasian, so these results will need to be replicated in more diverse study populations as we could not make inferences regarding any potential differences in results that may have been caused by differences in race.

The methodology of a 4-spot urine collection on filter paper followed by GC–MS/MS or LC–MS/MS offers some advantages. The collection of dried urine on filter paper results in stable measurement of steroid hormones for extended periods of time both by us [11] and others [12] for up to one year [13], even at ambient temperature. Concentrations of organic acids are also stable on dried filter paper for weeks [32, 33]. Mass spectrometry assays, which are now the gold standard for measurement of steroid hormones in blood and urine [10], allow for the use of small sample volumes with excellent sensitivity and accuracy along with simultaneous measurement of a relatively large number of analytes. In combination with chromatography, either gas or liquid, it provides precise separation of closely related molecules [34] by their chemical and physical properties. GC–MS/MS does not exclude any lipophilic steroids, and so a run will contain all excreted steroids [9]. The use of the *H. pomatia* enzymes adds to the accuracy of the quantification of the hormone conjugates, as these enzymes include both a sulfatase and a glucuronidase. The method of GC–MS/MS does require an additional extraction and derivatization step, but workflows can be optimized to maximize throughput. A 24-h urine collection may be difficult for some patients to fully collect, especially if they are not able to remain at home for an entire day, are disabled, or are incontinent. This methodology removes that barrier and provides the ability to measure multiple hormones at once with a noninvasive collection method, obtaining a complete picture of both production and clearance of the major steroidal hormones.

A multitude of uses, both in research and in clinical scenarios, could be envisioned for assays that are able to measure multiple steroid hormones and organic acids in conveniently collected urine samples on filter paper. For example, the full range of hormonal changes in individuals related to disruption of the natural sleep cycle could be evaluated simultaneously. It is already known that the peak 6-sulfatoxymelatonin, as representative of melatonin, is lower in people working the night shift [35]; a full appreciation of the urinary steroid profile in individuals who work at night could add to this prior research. Similarly, urine profiling may help to fully define the changes expected in genetic syndromes of steroidogenesis [5, 36] and errors of metabolism [16]. Dried urine samples may be of particular benefit in screening neonates for organic acid disorders [15, 16], and there is recent interest in a possible association of organic acids with neuropsychiatric disorders [37–39]. The ability to look at a full urine profile can provide a more integrated view of the patient; for example, patients using oral contraceptives often have higher xanthurenic acid with concurrent pyroxidine deficiency [40], both of which would be observable using dried urine analysis. A greater understanding of the full effect of changes in hormonal concentrations and metabolites or important clinical subgroups could be determined for both exogenous use of hormones and for exposure to endocrine disrupting compounds like bisphenol A [41, 42]. Urine hormone profiling might also be used to fully describe age related changes, i.e. through puberty or menopause.

## Conclusions

Mass spectrometry allows for the assessment of a full hormone profile in a small volume of urine such that an expanded view of both hormone production and clearance can be observed. In addition, results from dried urine are in strong agreement with those obtained from liquid urine. In combination with four spot urine collections on filter paper collected throughout the waking hours, we have shown that it is possible to accurately represent a 24-h urine collection. This technology may be useful to the clinician wishing to perform a large series of tests on patients to narrow the differential diagnosis, for those monitoring hormonal therapy or evaluating the menstrual cycle, or for those who need to reduce the burden of collection for their patients. This four-spot, dried urine method allows for assessment of both diurnal patterns [14] as well as total daily production, allowing for a comprehensive evaluation of adrenal and reproductive hormones and other urine metabolites.

## Supplementary Information

**Additional file 1. Table S1:** Partial list of urine hormones and analytes that can be measured from dried urine.** Figure S1:** Correlations between the liquid versus dried measurements for selected urine hormones.** Figure S2:** Correlations between the liquid versus dried measurements for selected urine organic acids.** Figure S3:** Correlations between the 24-h urine collection and 4-spot (DUTCH) urine collection measurements.** Figure S4:** Correlations between the 24-h urine collection and the 4-spot (DUTCH) urine collection estradiol measurements with (C) and without the two creatinine (Cr) corrections (A & B).

## Data Availability

The datasets acquired and/or analyzed during the current study are available from the corresponding author on reasonable request.

## Abbreviations

2OHE1: 2-Hydroxyestrone
2-methoxyE1: 2-Methoxyestrone
4OHE1: 4-Hydroxyestrone
5-HIAA: 5-Hydroxyindoleacetic acid
16OHE1: 16-Hydroxyestrone
CAN: Acetonitrile
CLIA: Clinical Laboratory Improvement Amendments
Cr: Creatinine
DHEA: Dehydroepiandrosterone
DHT: 5⍺-Dihydrotestosterone
DUTCH: Dried Urine Testing for Comprehensive Hormones
E1: Estrone
E2: Estradiol
E3: Estriol
EpiT: Epitestosterone
GC–MS/MS: Gas chromatography with tandem mass spectrometry
Hiv: β-Hydroxyisovaleric
HVA: Homovanillic acid
ICC: Intraclass correlation coefficient
IQR: Interquartile range
LC–MS/MS: Liquid chromatography with tandem mass spectrometry
MMA: Methylmalonic acid
T: Testosterone
TQD: Tandem quadrupole mass spectrometer detector
VMA: Vanillylmandelic acid
