# Supplementary material for: Reliability of a dried urine test for comprehensive assessment of urine hormones and metabolites
Source: BMC Chem. 2021 Mar 15;15(1):18. doi: 10.1186/s13065-021-00744-3 (PMC7962249; doi:10.1186/s13065-021-00744-3)
Supplement: Supplementary file 1 — Additional file 1. Table S1: Partial list of urine hormones and analytes that can be measured from dried urine. Figure S1: Correlations between the liquid versus dried measurements for selected urine hormones. Figure S2: Correlations between the liquid versus dried measurements for selected urine organic acids. Figure S3: Correlations between the 24-h urine collection and 4-spot (DUTCH) urine collection measurements. Figure S4: Correlations between the 24-h urine collection and the 4-spot (DUTCH) urine collection estradiol measurements with (C) and without the two creatinine (Cr) corrections (A & B). [file 13065_2021_744_MOESM1_ESM.docx]

**Supplementary Material**

**Title**

Reliability of A Dried Urine Test for Comprehensive Assessment of Urine Hormones and Metabolites

**Authors**

Mark Newman, Precision Analytical, Inc. 3138 NE Rivergate Street #301C, McMinnville, OR 97128 USA, mnewman@dutchtest.com (Corresponding author)

Desmond A. Curran, Precision Analytical, Inc. 3138 NE Rivergate Street #301C, McMinnville, OR 97128 USA, [dcurran@dutchtest.com](mailto:dcurran@dutchtest.com)

**Figure titles and legends for supplemental files**

**Supplemental Figure 1.** Correlations between the liquid versus dried measurements for selected urine hormones. Reported correlation coefficients are Spearman correlations.

Cr = creatinine, OH = hydroxy

**Supplemental Figure 2.** Correlations between the liquid versus dried measurements for selected urine organic acids. Reported correlation coefficients are Spearman correlations. Cr=creatinine, 5-HIAA = 5-hydroxyindoleacetic acid

**Supplemental Figure 3.** Correlations between the 24-h urine collection and 4-spot (DUTCH) urine collection measurements. Reported correlation coefficients are Spearman correlations.

**Supplemental Figure 4.** Correlations between the 24-h urine collection and the 4-spot (DUTCH) urine collection estradiol measurements with (C) and without the two creatinine (Cr) corrections (A & B). The traditional creatinine correction (B) is simply dividing by the amount of creatinine in the sample. The full correction (A) also accounts for differences in expected creatinine excretion based on body size. Reported correlation coefficients are Spearman correlations.

**Supplementary table 1.** Partial list of urine hormones and analytes that can be measured from dried urine

DHEA = dehydroepiandrosterone

| **Variable** | **Pathway** | **Examples of Clinical Relevance** |
| --- | --- | --- |
| **Estrone** | Estrogen; estradiol metabolite | Monitoring of hormonal replacement therapy; assessment of ovarian function or precocious puberty; evaluation of dietary effects^1–5^ |
| **Estradiol** | Primary active estrogen | Investigation of amenorrhea; assessment of precocious puberty; monitoring of hormonal replacement therapy; evaluation of dietary effects^1,5–8^ |
| **Estriol** | Estrogen; estrogen metabolite via 16α-hydroxyestrone | Monitoring of hormonal replacement therapy; evaluation of polycystic ovarian syndrome (PCOS); evaluation of dietary effects^1,5,9^ |
| **2-hydroxyestrone** | Estrogen metabolite via estrone | Estrogen receptor antagonist; antiproliferative; relative to other metabolites, may indicate health risks^6,7,10,11^ |
| **2-hydroxyestradiol** | Estrogen metabolite via 2-hydroxyestrone | Antiproliferative; relative to other metabolites, may indicate health risks; potential placental antioxidant^12^ |
| **4-hydroxyestrone** | Estrogen metabolite via estrone | Estrogen receptor agonist; relative to other metabolites, may indicate health risks; may be proangiogenic^11^ |
| **16-hydroxyestrone** | Estrogen metabolite via estrone | Estrogen receptor agonist; proliferative; relative to other metabolites, may indicate health risks; 2/16OHE1 ratio may be a marker of estrogen related cancer risk; together with estriol indicates activity of 16-hydroxylation pathway^6,7,10,11^ |
| **2-methoxyestrone** | Estrogen metabolite via 2-hydroxyestrone | May be anti-angiogenic; together with 2-hydroxyestrone and 2-hydroxyestradiol indicates activity of 2-hydroxylation pathway^13,14^ |
| **Testosterone** | Primary active androgen | Evaluation of male hypogonadism and infertility; monitoring of hormone therapy; anabolic to muscle and bone; evaluation of PCOS^1,15–18^ |
| **Epitestosterone** | Inactive epimer of testosterone | Evaluation of exogenous testosterone use; weak androgen antagonist^15,18^ |
| **5α-Dihydrotestosterone** | Active form of testosterone in tissues | Potent androgen; evaluation of hirsutism; evaluation of male infertility, hypogonadism, and low libido^15,19^ |
| **Androsterone** | Metabolite of testosterone via 5αDHT | Weak androgenic activity; neurosteroid; primary androgen metabolite in urine; evaluation of male infertility, hypogonadism, low libido, and hormone therapy; evaluation of PCOS^15,20^ |
| **Etiocholanolone** | 5β epimer of androsterone | Primary androgen metabolite in urine; inhibitory neurosteroid; evaluation of male infertility, hypogonadism, low libido, and hormone therapy; evaluation of PCOS^15,20^ |
| **5α-Androstanediol** | Metabolite of testosterone and 5αDHT | Weak androgenic and estrogenic properties; inhibitory neurosteroid; involved in prostate growth; evaluation of male infertility, hypogonadism, and low libido; evaluation of PCOS^9,15^ |
| **5β-Androstanediol** | Metabolite of testosterone and 5αDHT; β epimer of 5α-androstanediol | Has estrogen agonist properties; evaluation of male infertility, hypogonadism, and low libido; evaluation of PCOS^9,15^ |
| **Dehydroepiandrosterone** **(DHEA)** | Major metabolite in urine is DHEAS | Weak androgen produced by adrenal glands and brain; neurosteroid; implicated in neurocognitive declines and low libido; assessment of hirsutism and age-related changes in women; assessment of subclinical hypercortisolism^19,21^ |
| **6-Hydroxymelatoninsulfate** | Metabolite of melatonin | Representative of melatonin which is involved in the regulation of the sleep-wake cycle and promotes sleep; neuroprotective; evaluation of insomnia, fatigue, and infertility^22,23^ |
| **Methylmalonic Acid** | Intermediate in the propionate pathway | Increased in thiamine (B12) deficiency^24^ |
| **Homovanillic Acid** | Metabolite of dopamine | Elevated in cases of neuroblastoma; marker of metabolic stress in patients with psychiatric and neurologic diseases^25^ |
| **Vanillylmandelic Acid** | Metabolite of norepinephrine | Elevated in cases of neuroblastoma or pheochromocytoma^25,26^ |
| **Kynurenic Acid** | Metabolite of tryptophan | Part of the kynureninase pathway; increased in pyridoxine (B6) deficiency^27–30^ |
| **Xanthurenic Acid** | Metabolite of tryptophan | Part of the kynureninase pathway; increased in pyridoxine (B6) deficiency; possible measure of oxidative stress^29–31^ |
| **5-hydroxyindoleacetic acid (5-HIAA)** | Metabolite of tryptophan | End product of serotonin metabolism; elevated in carcinoid syndrome; reduced in patients with major depressive disorder^25,32,33^ |
| **Pyroglutamic Acid** | Intermediate in glutathione metabolism | Increased in glutathione (an antioxidant) deficiency, as can occur with excess acetaminophen use^34,35^ |
| **β-Hydroxyisovaleric Acid** | Metabolite of leucine | Increased in biotin (B7) deficiency and in smokers^36,37^ |

**Supplemental Figure 1**

**Supplemental Figure 2**

**Supplemental Figure 3**

**Supplemental Figure 4**
